# Relation of Serum α- and γ-Tocopherol Levels to Cardiovascular Disease-Related Mortality Among Japanese Men and Women

**DOI:** 10.2188/jea.JE20120002

**Published:** 2012-09-05

**Authors:** Masanori Nagao, Yuri Moriyama, Kazumasa Yamagishi, Hiroyasu Iso, Akiko Tamakoshi

**Affiliations:** 1 Public Health, Department of Social and Environmental Medicine, Osaka University Graduate School of Medicine, Suita, Osaka, Japan; 1 大阪大学大学院医学系研究科公衆衛生学; 2 Department of Public Health, Dokkyo Medical University School of Medicine, Shimotsugagun, Tochigi, Japan; 2 獨協医科大学公衆衛生学講座; 3 Public Health Institute of Kochi Prefecture, Kochi, Japan; 3 前高知県衛生研究所; 4 Department of Public Health Medicine, Faculty of Medicine, University of Tsukuba, Tsukuba, Ibaraki, Japan; 4 筑波大学大学院人間総合科学研究科社会健康医学; 5 Department of Public Health, Aichi Medical University School of Medicine, Nagakute, Aichi, Japan; 5 愛知医科大学医学部公衆衛生学教室

**Keywords:** α-tocopherol, γ-tocopherol, vitamin E, prospective study, stroke, cardiovascular disease, nested case-control study

## Abstract

**Background:**

There is limited evidence regarding the relationship between serum tocopherol levels and cardiovascular disease.

**Methods:**

We conducted a nested case-control study as part of the Japan Collaborative Cohort Study for evaluation of cancer risk (JACC Study). Baseline serum samples were collected from 39 242 participants (age range, 40–79 years) between 1988 and 1990. During the 13-year follow-up, there were 530 stroke deaths (302 ischemic strokes and 210 hemorrhagic strokes) and 211 deaths from coronary heart disease. Controls were matched for sex, age, and area of residence.

**Results:**

Serum α-tocopherol level was not associated with any type of cardiovascular death in men; however, in women, it was inversely associated with total stroke mortality and hemorrhagic stroke mortality. The multivariate odds ratio (95% CI) for the highest versus the lowest quintile of serum α-tocopherol levels among women was 0.35 (0.16–0.77; *P* for trend = 0.009) for total stroke and 0.26 (0.07–0.97; *P* for trend = 0.048) for hemorrhagic stroke. Serum γ-tocopherol was inversely associated with ischemic stroke mortality in men but positively associated with hemorrhagic stroke mortality in women. The respective multivariate odds ratios (95% CI) for the highest versus the lowest quintile and for a 1-standard deviation increment in γ-tocopherol level were 0.48 (0.22–1.06; *P* for trend = 0.07) and 0.77 (0.58–1.02), respectively, for ischemic stroke in men and 3.10 (0.95–10.12; *P* for trend = 0.052) and 1.49 (1.04–2.13) for hemorrhagic stroke in women.

**Conclusions:**

Among women, hemorrhagic stroke mortality was inversely associated with serum α-tocopherol and positively associated with serum γ-tocopherol. These findings are due in part to the antioxidative and antithrombotic activities of these tocopherols.

## Abstract (ja)

**【背景】:**

血清トコフェロール濃度と循環器疾患死亡リスクとの関連についての研究は非常に少ない。本研究では血清α-及びγ-トコフェロール濃度と脳卒中・虚血性心疾患死亡との関連を検証することを目的とした。

**【方法】:**

文部科学省助成大規模コホート（JACC Study）の1988－1990年のベースライン調査で血清提供に同意し、脳卒中、心疾患、がんの既往がない40－79歳の男女38,158人（男性13,382人、女性24,776人）を対象として、脳卒中死亡及び虚血性心疾患死亡をアウトカムとするコホート内症例対照研究を行った。対照は、ケースと性・年齢（±5歳）・居住地域・血清保管年数がマッチした者から無作為に抽出した。

**【結果】:**

13年間の追跡期間中、脳梗塞死亡302人（男性165人、女性137人）、脳出血死亡210人（男性85人、女性125人）、虚血性心疾患死亡211人（男性114人、女性97人）が認められた。血清α-トコフェロール濃度は男性においていずれの死亡とも関連がみられなかったが、女性では全脳卒中死亡と脳出血死亡との間に負の関連がみられた。女性の血清α-トコフェロール濃度最低値群に対する最高値群の多変量調整死亡オッズ比は、全脳卒中死亡が0.35（0.16-0.77; *P* for trend = 0.009）、脳出血死亡が0.26（0.07-0.97; *P* for trend = 0.048）であった。血清γ-トコフェロール濃度は男性の脳梗塞死亡と負の関連が見られたが、女性の脳出血死亡とは正の関連がみられた。最低値群に対する最高値群、及び1標準偏差増加分における多変量調整オッズ比は、男性脳梗塞死亡が0.48（0.22–1.06; p for trend = 0.07）及び0.77（0.58–1.02）、女性脳梗塞死亡が3.10（0.95-10.12; *P* for trend = 0.052）及び1.49（1.04-2.13）であった。

**【考察】:**

女性の脳出血死亡との関連において、血清α-トコフェロール濃度は負の関連を示したが、γ-トコフェロール濃度は正の関連を示した。これらの結果にはトコフェロールの抗酸化作用と抗血栓作用が関与していることが考えられる。

## INTRODUCTION

Tocopherols are believed to be promising agents for reducing the risk of cardiovascular disease, due of their strong antioxidant activity.^1^ Although randomized control trials (RCTs) of tocopherol supplementation have been conducted,^2^^–^^7^ the results have been inconsistent. Several, but not all, RCTs have shown a beneficial effect of α-tocopherol supplementation on the risk of nonfatal myocardial infarction,^2^ subarachnoid hemorrhage,^3^ ischemic stroke,^3^ hemorrhagic stroke,^4^ and cardiovascular disease mortality.^5^ RCTs are useful for establishing causality, especially in evaluations of the short- and moderate-term effects of drugs. However, it is difficult to implement RCTs of the effects of long-term dietary exposure. Thus, observational studies are a useful alternative.

Previous observational studies reported an association between intake of tocopherol, either from food or via supplementation, and cardiovascular disease risk.^8^^–^^12^ However, pharmacokinetic studies suggest that complex mechanisms are involved in the regulation of tocopherol levels.^13^ For example, large consumption of vitamin E induces excretion of vitamin E via the pregnane X receptor drug metabolism system activated by vitamin E.^14^^–^^16^ In contrast, some nutrients, such as sesamin, increase blood tocopherol levels by suppressing tocopherol metabolism.^17^ Thus, tocopherol intake is not always reflected in serum tocopherols levels. For these reasons, we estimated serum levels of tocopherols and examined their relationship with cardiovascular disease risk. Although previous studies have examined the relationship of serum levels of α- and γ-tocopherol with cardiovascular disease risk in men,^18^^–^^22^ no such studies have been conducted in women.

In this nested case-control study using a large prospective cohort of approximately 40 000 men and women, we evaluated the association of serum levels of α- and γ-tocopherol with the risk of cardiovascular disease mortality among Japanese men and women.

## METHODS

### Survey population

We conducted a nested case-control study as part of the Japan Collaborative Cohort (JACC) Study. The methods of the JACC Study have been previously described. In brief, 110 792 individuals (46 465 men and 64 327 women) aged 40 to 79 years during the baseline period (1988–1990) were enrolled from 45 communities throughout Japan. Using self-administered questionnaires, participants gave information on their lifestyle and medical histories of cardiovascular disease and cancer.^23^^,^^24^

Written or explicit verbal informed consent to participate in the study was obtained from the individuals who completed the questionnaire. In several communities, informed consent was obtained at a community level after the purpose and methods of the study and its emphasis on data confidentiality had been explained to community leaders and mayors on behalf of the individual participants. At the time of recruitment for this study, this was a common method of obtaining informed consent in Japan.

A total of 39 242 participants (35.4% of the questionnaire respondents) agreed to provide blood samples and gave individual informed consent.^25^^,^^26^ After exclusion of 457 men and 627 women with a history of heart disease, stroke, or cancer at the baseline survey, a total of 38 158 participants (13 382 men and 24 776 women) were enrolled in the present study. The ethics committees of the University of Tsukuba and Osaka University approved the present study.

### Mortality surveillance

The participants were followed up to determine mortality due to cardiovascular disease until the end of 2003, except for 4 communities in which follow-up ended in 1999.

The study investigators conducted a systematic review of death certificates in each community. Because registration of death is a legal requirement throughout Japan, the investigators were confident of completing the follow-up. Participants who had moved out of their original community were treated as censored cases.

To determine cause-specific mortality of cardiovascular disease, we used the International Classification of Diseases (ICD), 9th and 10th revisions, as follows: total stroke (ICD 9th revision, codes 430–438 and ICD 10th revision, codes I60–I69); coronary heart disease (410–414, I20–I25). Total stroke was subdivided into hemorrhagic stroke (430–431, I60–I61) and ischemic stroke (433–434, I63). For each case, we randomly selected 1 control participant (ie, a person without death from stroke or coronary heart disease) from the participants. Controls were matched for sex, age (±5 years), community, tocopherol measurement method, and year of blood drawing.

### Determination of biochemical variables

Venous blood was collected at baseline, and sera were prepared from blood samples as soon as possible after blood collection at laboratories in or near the surveyed municipalities. The serum samples were collected in 0.3-ml tubes and stored at −80°C until further use. Serum α-tocopherol and γ-tocopherol levels were measured using high-performance liquid chromatography (HPLC) at the Osaka laboratory facility of SRL, Inc. (Tokyo, Japan; 246 case-control pairs) and the Public Health Institute of Kochi Prefecture (495 case-control pairs). The analytical protocols for tocopherol estimation were similar at both facilities and included the deproteinization of serum samples, extraction of tocopherols by an organic solvent, and assay by reversed-phase HPLC using a fluorescent detector. However, there were some differences between the SRL and Kochi methods. The SRL method involved deproteinization by ethanol, extraction by n-hexane, and concentration using nitrogen gas, whereas the Kochi institute method involved deproteinization and extraction by 2-propanol, but no concentration step.

The measurements obtained at SRL were adjusted using the results of 30 samples that were measured by both methods. The regression equations for the values obtained by the 2 methods were y_1_ = 2.135x_1_ − 1.036 and y_2_ = 1.483x_2_ + 0.203 for α-tocopherol and γ-tocopherol, respectively, where y_1_ and y_2_ were Kochi method values and x_1_ and x_2_ were SRL method values. The correlation coefficients were 0.903 and 0.970 for α-tocopherol and γ-tocopherol, respectively.

Serum total cholesterol was measured by an enzymatic method using an automatic analyzer at Kotobiken Medical Laboratories, Inc. (Tokyo, Japan). The standardization of lipid measurement was performed with the aid of the Osaka Medical Center for Health Science and Promotion, which is an international member of the US National Cholesterol Reference Method Laboratory Network (CRMLN).^27^

### Statistical analysis

Because tocopherols are highly correlated with total cholesterol levels, we used the residual method to adjust α- and γ-tocopherol levels for serum total cholesterol.^28^

To compare the baseline characteristics of mortality cases and control subjects, the paired *t*-test was used to test mean values, and the McNemar test was used for percentages of cardiovascular risk factors.

Cut-off values in quintile analyses of the distribution of control subjects were determined. Using conditional logistic regression models, odds ratios (ORs) for total stroke, stroke subtype, and coronary heart disease were estimated according to sex-specific quintiles of serum α- and γ-tocopherol levels and 1-SD increments in α-tocopherol levels (5.12 µg/ml for men and 6.90 µg/ml for women) and γ-tocopherol levels (0.61 µg/ml for men and 0.67 µg/ml for women). Linear regression was used to test for linear trends across tocopherol categories by using the median tocopherol level for each tocopherol category. Covariates for adjustment included body mass index (categorized as <18.5 kg/m^2^, 18.5–24.9 kg/m^2^, and ≥25 kg/m^2^), serum total and high-density lipoprotein (HDL) cholesterol levels (mg/dl), cigarette smoking status (never, former, current), alcohol drinking status (never, ex-, and current), walking (≥30 min/day or not), sports (≥5 hour/week or not), and self-reported history of a physician diagnosis of hypertension or diabetes mellitus (yes or no), as well as matching for sex, age, area of residence, tocopherol measurement method, and year of blood drawing.

The data were analyzed with Statistical Analysis Software (SAS; Version 9.1.3 Service Pack 4; SAS Institute, Cary, NC, USA). All probability values for statistical tests were 2-tailed, and a *P* value less than 0.05 was considered statistically significant.

## RESULTS

During the 13-year follow-up, we documented 530 stroke deaths (257 men and 273 women). Of these, 302 deaths (165 men and 137 women) were due to ischemic stroke and 210 (85 men and 125 women) were due to hemorrhagic stroke, whereas 211 deaths (114 men and 97 women) were due to coronary heart disease.

Table 1 shows the sex-specific characteristics of cases and controls. Mean age at death was 67 years among men and 72 years among women for ischemic stroke, 63 years among men and 64 years among women for hemorrhagic stroke, 64 years among men and 69 years among women for coronary heart disease. Among cases compared with controls, the prevalence of hypertension was higher in men who had any stroke, ischemic stroke, or coronary heart disease and in women with hemorrhagic stroke; average alcohol intake and prevalence of current smoking were higher in men; the prevalence of current smoking was higher in men with any stroke or ischemic stroke; the prevalence of diabetes mellitus was higher in women with coronary heart disease; mean serum total cholesterol was lower in women with any stroke or hemorrhagic stroke; and mean serum α-tocopherol tended to be lower for all cardiovascular endpoints among both men and women, except for hemorrhagic stroke in men.

**Table 1.** Sex-specific mean values or prevalence of cardiovascular risk factors in cases and controls

	Total stroke		Ischemic stroke		Hemorrhagic stroke		Coronary heart disease	
				
	Cases	Controls	Cases	Controls	Cases	Controls	Cases	Controls
**Men**								
No. of subjects	257	257	165	165	85	85	114	114
Age, years	66.6 ± 0.5	65.8 ± 0.5	67.9 ± 0.6	67.2 ± 0.5	64.1 ± 1.0	63.1 ± 0.9	64.7 ± 1.0	64.0 ± 0.9
Hypertension, %	40.5***	23.7	43.6***	24.8	32.9	21.2	36.0*	21.1
Diabetes mellitus, %	7.0	5.4	8.5	6.1	4.7	4.7	9.6	7.9
BMI, kg/m^2^	22.3 ± 0.2	22.3 ± 0.2	22.2 ± 0.2	22.1 ± 0.2	22.2 ± 0.3	22.6 ± 0.3	22.7 ± 0.3	22.7 ± 0.3
Alcohol intake, g/day	20.7 ± 1.6*	16.6 ± 1.4	18.2 ± 1.7	16.2 ± 1.7	26.3 ± 3.2	18.4 ± 2.7	17.7 ± 2.2	15.5 ± 2.1
Current smoker, %	56.1*	45.9	57.4*	46.8	54.2	42.9	53.2	49.1
Total cholesterol, mg/dl	184.1 ± 2.4	190.1 ± 2.2	187.0 ± 2.9	189.8 ± 2.7	179.4 ± 4.6	189.6 ± 4.0	196.4 ± 3.5	194.2 ± 3.6
HDL cholesterol, mg/dl	44.6 ± 1.0	46.1 ± 1.0	44.6 ± 1.2	47.5 ± 1.3	44.5 ± 1.8	44.3 ± 1.7	44.9 ± 1.5	44.2 ± 1.3
Walking ≥0.5 h/week, %	68.3	77.7	68.4	76.7	70.5	79.0	66.2	66.7
Sports ≥5 h/week, %	10.2	12.4	10.2	11.5	9.4	14.7	11.4	7.3
Vitamin E intake, mg	5.9 ± 0.2	5.6 ± 0.2	6.1 ± 0.2	5.7 ± 0.2	5.6 ± 0.3	5.5 ± 0.3	5.1 ± 0.3	4.8 ± 0.3
Serum α-tocopherol, µg/ml	13.67 ± 0.33	13.78 ± 0.33	13.77 ± 0.43	14.04 ± 0.42	13.57 ± 0.56	13.27 ± 0.55	13.17 ± 0.41	13.51 ± 0.47
Serum γ-tocopherol, µg/ml	1.08 ± 0.04	1.17 ± 0.04	1.04 ± 0.04	1.14 ± 0.05	1.18 ± 0.06	1.21 ± 0.06	1.29 ± 0.07	1.20 ± 0.06
**Women**								
No. of subjects	273	273	137	137	125	125	97	97
Age, years	68.3 ± 0.6	68.1 ± 0.5	71.9 ± 0.7	71.5 ± 0.6	64.2 ± 0.8	64.2 ± 0.8	68.9 ± 0.8	68.7 ± 0.8
Hypertension, %	40.3	32.2	35.8	36.5	43.2**	26.4	40.2	39.2
Diabetes mellitus, %	5.9	4.0	7.3	5.1	4.8	2.4	17.5**	4.1
BMI, kg/m^2^	22.4 ± 0.2	22.9 ± 0.2	22 ± 0.3	22.4 ± 0.3	22.8 ± 0.3	23.2 ± 0.3	23.6 ± 0.4	23.2 ± 0.3
Alcohol intake, g/day	1.1 ± 0.4	1.0 ± 0.3	0.6 ± 0.3	1.1 ± 0.4	1.7 ± 0.9	0.7 ± 0.2	2.2 ± 1.1	0.9 ± 0.5
Current smoker, %	5.6	3.3	7.1	4.2	4.5	2.6	18.2	9.0
Total cholesterol, mg/dl	204.2 ± 2.6**	214.2 ± 2.5	205.7 ± 4.1	213.1 ± 3.5	202.3 ± 3.5*	214.8 ± 3.7	217.5 ± 4.9	213.6 ± 4.3
HDL cholesterol, mg/dl	44.0 ± 0.9	45.1 ± 0.9	45.0 ± 1.3	45.3 ± 1.3	43.1 ± 1.2	44.7 ± 1.3	39.9 ± 1.4*	44.5 ± 1.7
Walking ≥0.5 h/week, %	70.8	75.3	71.7	75.5	73.0	73.1	67.2	76.2
Sports ≥5 h/week, %	5.9	9.1	6.7	11.5	5.5	6.3	1.4	7.9
Vitamin E intake, mg	5.4 ± 0.2	5.3 ± 0.1	5.4 ± 0.2	5.3 ± 0.2	5.4 ± 0.2	5.3 ± 0.2	5.0 ± 0.3	5.4 ± 0.2
Serum α-tocopherol, µg/ml	15.63 ± 0.38	16.21 ± 0.35	15.40 ± 0.57	16.06 ± 0.46	15.78 ± 0.48	16.57 ± 0.56	15.34 ± 0.58	16.46 ± 1.15
Serum γ-tocopherol, µg/ml	1.33 ± 0.04	1.33 ± 0.04	1.28 ± 0.05	1.38 ± 0.05	1.41 ± 0.06	1.27 ± 0.06	1.32 ± 0.08	1.43 ± 0.07

Abbreviations: BMI, body mass index; HDL, high-density lipoprotein.

Serum α- and γ-tocopherol values were adjusted for total cholesterol by using the residual method.

Difference from controls: *P < 0.05, **P < 0.01, ***P < 0.001.

In addition, among cases, mean serum γ-tocopherol tended to lower for all cardiovascular endpoints among both men and women, except for coronary heart disease in men and any stroke and hemorrhagic stroke in women.

Table 2 shows age-, sex-, and community-matched ORs and multivariate ORs (95% CIs) for cardiovascular disease mortality according to quintile of serum α-tocopherol levels. In men, there was no association between serum α-tocopherol and cardiovascular disease mortality. In women, serum α-tocopherol was inversely associated with mortality from any stroke and hemorrhagic stroke. The multivariate OR (95% CI) for the highest versus lowest quintile of serum α-tocopherol among women was 0.35 (0.16–0.77; *P* for trend = 0.009) for total stroke, 0.47 (0.14–1.52; *P* for trend = 0.12) for ischemic stroke, 0.26 (0.07–0.97; *P* for trend = 0.048) for hemorrhagic stroke, and 0.97 (0.24–3.95; *P* for trend = 0.85) for coronary heart disease.

**Table 2.** Sex-specific odds ratios (ORs) and 95% CIs for cardiovascular disease mortality by quintile of total cholesterol-adjusted serum α-tocopherol

		Serum α-tocopherol level								*P* for trend	1-SD increment
		
	Q1 (low)	Q2		Q3		Q4		Q5 (high)			
					
		OR	95% CI	OR	95% CI	OR	95% CI	OR	95% CI		
**Men**											
Serum α-tocopherol											
Median, µg/ml	8.7	11.1		12.8		14.7		19.5			
Range, µg/ml	(1.7–10.2)	(10.2–11.8)		(11.8–13.7)		(13.7–16.3)		(16.3–49.0)			
Total stroke											
No. of cases	63	32		56		50		56			
No. of controls	52	52		48		52		53			
Age-, community-matched OR	1.00	0.50	(0.27–0.91)	0.93	(0.51–1.70)	0.80	(0.43–1.49)	0.92	(0.49–1.70)	0.92	0.98 (0.81–1.17)
Multivariable OR^a^	1.00	0.44	(0.22–0.86)	0.85	(0.44–1.63)	0.73	(0.37–1.42)	0.81	(0.41–1.60)	0.87	0.91 (0.75–1.11)
Ischemic stroke											
No. of cases	38	22		37		30		38			
No. of controls	31	28		30		43		33			
Age-, community-matched OR	1.00	0.63	(0.30–1.33)	0.92	(0.42–1.98)	0.48	(0.21–1.09)	0.84	(0.38–1.85)	0.89	0.95 (0.76–1.18)
Multivariable OR^a^	1.00	0.63	(0.26–1.52)	0.90	(0.38–2.16)	0.46	(0.19–1.14)	0.83	(0.35–2.00)	0.91	0.90 (0.71–1.15)
Hemorrhagic stroke											
No. of cases	23	9		17		19		17			
No. of controls	19	24		15		9		18			
Age-, community-matched OR	1.00	0.23	(0.07–0.78)	0.97	(0.34–2.75)	1.58	(0.55–4.59)	0.89	(0.29–2.74)	0.61	1.08 (0.76–1.52)
Multivariable OR^a^	1.00	0.22	(0.05–0.95)	1.83	(0.47–7.11)	2.11	(0.54–8.18)	0.97	(0.25–3.77)	0.58	1.09 (0.74–1.61)
Coronary heart disease											
No. of cases	28	23		22		20		21			
No. of controls	21	23		26		23		21			
Age-, community-matched OR	1.00	0.76	(0.34–1.70)	0.63	(0.28–1.42)	0.63	(0.26–1.51)	0.71	(0.29–1.78)	0.50	0.91 (0.66–1.25)
Multivariable OR^a^	1.00	0.78	(0.31–1.94)	0.59	(0.24–1.45)	0.65	(0.23–1.83)	0.68	(0.24–1.92)	0.46	0.87 (0.61–1.25)
**Women**											
Serum α-tocopherol											
Median, µg/ml	9.6	13.0		15.3		17.9		23.0			
Range, µg/ml	(0.0–11.8)	(11.8–14.1)		(14.2–16.2)		(16.2–19.9)		(19.9–103.8)			
Total stroke											
No. of cases	68	57		45		61		42			
No. of controls	52	52		56		53		60			
Age-, community-matched OR	1.00	0.70	(0.39–1.25)	0.48	(0.26–0.92)	0.61	(0.32–1.20)	0.39	(0.20–0.78)	0.01	0.86 (0.68–1.08)
Multivariable OR^a^	1.00	0.70	(0.37–1.34)	0.44	(0.21–0.91)	0.52	(0.24–1.10)	0.35	(0.16–0.77)	0.009	0.85 (0.66–1.10)
Ischemic stroke											
No. of cases	37	31		20		27		22			
No. of controls	30	19		31		25		32			
Age-, community-matched OR	1.00	1.05	(0.43–2.60)	0.36	(0.14–0.95)	0.53	(0.19–1.48)	0.37	(0.14–1.02)	0.03	0.83 (0.59–1.16)
Multivariable OR^a^	1.00	1.19	(0.40–3.57)	0.39	(0.12–1.29)	0.51	(0.15–1.74)	0.47	(0.14–1.52)	0.12	0.97 (0.66–1.43)
Hemorrhagic stroke											
No. of cases	25	25		25		34		16			
No. of controls	19	31		23		25		27			
Age-, community-matched OR	1.00	0.57	(0.25–1.32)	0.76	(0.30–1.91)	0.87	(0.34–2.23)	0.38	(0.14–1.09)	0.15	0.81 (0.58–1.15)
Multivariable OR^a^	1.00	0.61	(0.22–1.74)	0.74	(0.22–2.46)	0.62	(0.18–2.05)	0.26	(0.07–0.97)	0.048	0.68 (0.44–1.05)
Coronary heart disease											
No. of cases	22	25		20		12		18			
No. of controls	21	23		17		21		15			
Age-, community-matched OR	1.00	0.99	(0.40–2.45)	1.01	(0.39–2.59)	0.52	(0.18–1.50)	0.99	(0.36–2.75)	0.88	0.89 (0.68–1.15)
Multivariable OR^a^	1.00	0.66	(0.18–2.33)	0.89	(0.25–3.17)	0.33	(0.08–1.32)	0.97	(0.24–3.95)	0.85	0.92 (0.62–1.36)

^a^Adjusted for body mass index (<18.5, 18.5–24.9, 25≤), cigarette smoking status (never, ex-, and current), alcohol drinking status (never, ex-, and current), history of hypertension and diabetes mellitus, walking (≥30 min/day or not), sports (≥5 h/week or not), total cholesterol, and high-density lipoprotein cholesterol (continuous).

In age-, sex-, and community-matched analyses, there was no significant association between serum γ-tocopherol and mortality from any cardiovascular disease outcome among men (Table 3). After adjusting for cardiovascular risk factors, the association between serum γ-tocopherol and ischemic stroke death was of borderline significance in men: the multivariate ORs (95% CI) for the highest versus the lowest quintile and for a 1-SD increment in γ-tocopherol level were 0.48 (0.22–1.06; *P* for trend = 0.07) and 0.77 (0.58–1.02), respectively. In women, serum γ-tocopherol was associated with increased hemorrhagic stroke mortality. The multivariate OR (95% CIs) for the highest versus the lowest quintile and for a 1-SD increment in γ-tocopherol level were 3.10 (0.95–10.1; *P* for trend = 0.052) and 1.49 (1.04–2.13), respectively.

**Table 3.** Sex-specific odds ratios (ORs) and 95% CIs for cardiovascular disease mortality by quintile of total cholesterol-adjusted serum γ-tocopherol

		Serum γ-tocopherol level								*P* for trend	1-SD increment
		
	Q1 (low)	Q2		Q3		Q4		Q5 (high)			
					
		OR	95% CI	OR	95% CI	OR	95% CI	OR	95% CI		
**Men**											
Serum γ-tocopherol											
Median, µg/ml	0.53	0.83		1.07		1.37		1.96			
Range, µg/ml	(0.01–0.70)	(0.71–0.95)		(0.95–1.21)		(1.21–1.60)		(1.60–4.53)			
Total stroke											
No. of cases	68	48		49		46		46			
No. of controls	52	47		52		56		50			
Age-, community-matched OR	1.00	0.78	(0.45–1.36)	0.69	(0.40–1.20)	0.59	(0.33–1.03)	0.66	(0.38–1.17)	0.15	0.84 (0.68–1.03)
Multivariable OR^a^	1.00	0.73	(0.40–1.34)	0.75	(0.41–1.37)	0.55	(0.30–1.03)	0.68	(0.37–1.25)	0.21	0.83 (0.67–1.04)
Ischemic stroke											
No. of cases	50	34		30		23		28			
No. of controls	37	31		33		32		32			
Age-, community-matched OR	1.00	0.84	(0.44–1.62)	0.64	(0.32–1.26)	0.50	(0.25–1.02)	0.60	(0.30–1.19)	0.12	0.80 (0.62–1.04)
Multivariable OR^a^	1.00	0.81	(0.38–1.69)	0.57	(0.26–1.23)	0.48	(0.21–1.09)	0.48	(0.22–1.06)	0.07	0.77 (0.58–1.02)
Hemorrhagic stroke											
No. of cases	16	14		18		19		18			
No. of controls	12	16		19		21		17			
Age-, community-matched OR	1.00	0.62	(0.21–1.84)	0.67	(0.24–1.89)	0.63	(0.21–1.85)	0.75	(0.26–2.23)	0.78	0.93 (0.66–1.31)
Multivariable OR^a^	1.00	0.34	(0.08–1.58)	0.58	(0.12–2.72)	0.27	(0.05–1.37)	0.88	(0.18–4.31)	0.73	1.03 (0.65–1.62)
Coronary heart disease											
No. of cases	20	20		25		23		26			
No. of controls	22	27		22		18		25			
Age-, community-matched OR	1.00	0.84	(0.34–2.11)	1.32	(0.50–3.45)	1.43	(0.57–3.59)	1.19	(0.47–3.00)	0.64	1.13 (0.89–1.44)
Multivariable OR^a^	1.00	1.02	(0.36–2.93)	1.54	(0.48–4.91)	1.72	(0.60–4.98)	1.54	(0.52–4.62)	0.48	1.20 (0.91–1.57)
**Women**											
Serum γ-tocopherol											
Median, µg/ml	0.57	0.99		1.30		1.60		2.21			
Range, µg/ml	(0.01–0.81)	(0.81–1.15)		(1.15–1.44)		(1.44–1.80)		(1.80–4.98)			
Total stroke											
No. of cases	54	69		42		53		55			
No. of controls	61	54		54		49		55			
Age-, community-matched OR	1.00	1.48	(0.87–2.51)	0.88	(0.50–1.54)	1.26	(0.71–2.23)	1.13	(0.64–1.97)	0.92	1.01 (0.84–1.22)
Multivariable OR^a^	1.00	1.32	(0.74–2.36)	0.87	(0.48–1.60)	1.21	(0.64–2.26)	1.26	(0.68–2.32)	0.56	1.06 (0.86–1.30)
Ischemic stroke											
No. of cases	29	36		20		29		23			
No. of controls	29	26		26		25		31			
Age-, community-matched OR	1.00	1.46	(0.67–3.17)	0.75	(0.34–1.66)	1.14	(0.52–2.50)	0.70	(0.31–1.58)	0.27	0.81 (0.61–1.08)
Multivariable OR^a^	1.00	1.19	(0.49–2.87)	0.72	(0.29–1.79)	1.00	(0.39–2.60)	0.82	(0.33–2.04)	0.59	0.84 (0.61–1.16)
Hemorrhagic stroke											
No. of cases	20	32		21		22		30			
No. of controls	29	26		26		21		23			
Age-, community-matched OR	1.00	1.76	(0.82–3.80)	1.15	(0.50–2.66)	1.55	(0.63–3.79)	1.99	(0.85–4.65)	0.19	1.26 (0.97–1.65)
Multivariable OR^a^	1.00	1.44	(0.52–4.00)	0.90	(0.30–2.68)	1.82	(0.63–5.27)	3.10	(0.95–10.1)	0.052	1.49 (1.04–2.13)
Coronary heart disease											
No. of cases	25	21		12		16		23			
No. of controls	12	20		20		26		19			
Age-, community-matched OR	1.00	0.52	(0.20–1.33)	0.25	(0.08–0.77)	0.31	(0.12–0.81)	0.56	(0.21–1.48)	0.24	0.85 (0.64–1.13)
Multivariable OR^a^	1.00	0.66	(0.19–2.28)	0.27	(0.06–1.21)	0.23	(0.06–0.82)	0.57	(0.15–2.23)	0.32	0.87 (0.59–1.28)

^a^Adjusted for body mass index (<18.5, 18.5–24.9, 25≤), cigarette smoking status (never, ex-, and current), alcohol drinking status (never, ex-, and current), history of hypertension and diabetes mellitus, walking (≥30 min/day or not), sports (≥5 h/week or not), total cholesterol, and high-density lipoprotein cholesterol (continuous).

## DISCUSSION

In this prospective, nested case-control study of Japanese men and women from the general population without diagnosed cardiovascular disease at enrolment, higher baseline serum α-tocopherol was associated with lower mortality from total stroke and hemorrhagic stroke among women. Serum γ-tocopherol tended to be associated with lower mortality from ischemic stroke among men and higher mortality from hemorrhagic stroke among women. There was no significant association between α- or γ-tocopherol and coronary heart disease mortality among men or women.

To our knowledge, only 3 studies have investigated the association between blood levels of tocopherols and cardiovascular disease risk among men^18^^–^^22^; however, have been no such studies of women. Our study is the first assessment of the relationship between serum tocopherol levels and cardiovascular disease risk in women.

The Alpha-Tocopherol, Beta-Carotene Cancer Prevention (ATBC) study found that serum α-tocopherol was associated with a decreased risk of ischemic stroke among men: the multivariate relative risk (95% CI) of ischemic stroke for the highest versus the lowest quartile of α-tocopherol levels was 0.70 (0.55–0.89).^18^ The ATBC study also reported that serum α-tocopherol was inversely associated with risk of intracerebral hemorrhage: the multivariate relative risk (95% CI) of intracerebral hemorrhage for the highest versus the lowest quintile of α-tocopherol levels was 0.45 (0.26–0.77).^18^

The Physicians’ Health Study (PHS) reported no association between serum α- or γ-tocopherol levels and ischemic stroke risk among men.^20^ Serum α-tocopherol was not associated with risk of myocardial infarction in PHS^21^ or The Multiple Risk Factor Intervention Trial,^22^ while γ-tocopherol was associated with increased risk of myocardial infarction in PHS: the multivariate relative risk (95% CI) for the highest versus the lowest quintile was 2.14 (1.18–3.87; *P* for trend = 0.01).^21^ In the present study, we did not observe an association between serum α- or γ-tocopherol and coronary heart disease mortality in men or women. These inconsistent findings regarding serum tocopherol levels and myocardial infarction may be due to a paradoxical effect of tocopherol on oxidation. Although tocopherols are known antioxidants, several in vivo studies have reported that excessive concentrations of tocopherol can cause oxidative stress, leading to lipid peroxidation mediated by the tocopherol radical.^29^

The antioxidant activity of α-tocopherol is a potential pathophysiologic mechanism for the inverse association between serum α-tocopherol and hemorrhagic stroke mortality. A Japanese autopsy study found that 11% of 101 intracerebral hemorrhage cases had cerebral amyloid angiopathy,^30^ and the prevalence of amyloid angiopathy tended to be higher in women (28.0%) than in men (18.3%).^31^ In a neuroimaging study in the United States, 2.2% of 460 subarachnoid hemorrhage patients aged 60 years or older had amyloid angiopathy.^32^ Deposits of the amyloid beta protein cause degeneration of smooth muscle cells of the vascular media.^33^ In a mice model of amyloid beta deposition, α-tocopherol supplementation in mice aged 5 to 13 months reduced amyloid beta protein levels and the area of cerebral amyloid deposits in brain tissue, as compared with a control group.^34^

Cerebral aneurysm can occur due to the development of chronic inflammation mediated by nuclear factor κ-light-chain-enhancer of activated B cells (NF-κB),^35^ which is induced by oxidative stress.^36^ Cerebral aneurysms in antioxidant-treated rats were 54% smaller than in controls, due to inhibition of NF-κB activity.^37^

The reasons for the absence of an association between α-tocopherol and mortality from total stroke and its subtypes among men in the present study are unknown, although the lower prevalence of amyloid angiopathy^31^ and lower proportion of subarachnoid hemorrhage^38^ among men as compared with women might partially explain this finding.

In addition to acting as antioxidants, tocopherols are also inhibitors of platelet aggregation and thrombus formation.^39^^,^^40^ In an animal experiment, time to thrombus formation was 25% longer in rats fed α-tocopherol and 58% longer in rats fed γ-tocopherol as compared with controls. Platelet aggregation was 16% lower in rats fed α-tocopherol and 43% lower in rats fed γ-tocopherol as compared with controls.^40^ In humans, α-tocopherol supplementation alone reduced serum γ-tocopherol levels,^41^ while a supplement with equal amounts of α- and γ-tocopherols raised plasma α- and γ-tocopherol levels.^42^ A supplement with mixed tocopherols (100 mg γ-, 40 mg δ-, and 20 mg α-tocopherol; corresponding to a 20 mg α-tocopherol equivalent) reduced platelet aggregation by 14% as compared with pre-supplementation platelet aggregation values. No change in platelet aggregation was observed in subjects receiving only an α-tocopherol supplement.^39^ This inhibitory effect of γ-tocopherol on platelet aggregation and thrombus formation could account for the association between serum γ-tocopherol and reduced ischemic stroke mortality, as well as the association between serum γ-tocopherol and increased hemorrhagic stroke mortality, observed in the present study.

The present study has several strengths. It is the first study to evaluate the prospective association between serum α- and γ-tocopherol and cardiovascular disease mortality in women and Japanese men from the general population. The observational design of our study had the advantage of allowing evaluation of long-term use of tocopherol supplements in relation to mortality. Because the participants in the present nested case-control study were recruited throughout Japan, our study results can be generalized to the entire Japanese population.

The present study has several potential limitations. First, serum samples were collected from only approximately 35% of the total participants. However, because there were no apparent differences in age-adjusted mean values or proportions of major cardiovascular risk factors between the participants who did and did not provide serum samples (data not shown), selection bias in our evaluation of the association between serum tocopherol levels and cardiovascular disease mortality is likely to be limited. Second, we did not examine the long-term stability of tocopherols in deeply frozen serum. However, a previous study found that tocopherol was stable for at least 15 years in serum stored at temperatures of −70°C or lower.^43^ Third, we used only mortality data as an endpoint, which may have led to misclassification of diagnoses. However, use of computed tomography scans has been widespread in Japan since the 1980s, even in local hospitals; thus, it is likely that our use of death certificate records to classify stroke and its subtypes was sufficiently accurate.^44^ However, approximately one-third of deaths categorized as coronary heart disease on death certificates were misdiagnosed, as demonstrated by validation studies.^45^ This discrepancy may have partly contributed to the lack of association between serum tocopherols levels and coronary heart disease mortality observed in the present study.

In conclusion, serum α-tocopherol was associated with lower mortality from total and hemorrhagic strokes in women. γ-Tocopherol tended to be associated with lower mortality from ischemic stroke in men and increased mortality from hemorrhagic stroke in women. No association was found between α- or γ-tocopherol and coronary heart disease mortality in men or women. Further studies are required to evaluate the effectiveness of vitamin E in the prevention of cardiovascular disease.

## ONLINE ONLY MATERIALS

Abstract in Japanese.
