# Supplementary material for: Relation of Serum α- and γ-Tocopherol Levels to Cardiovascular Disease-Related Mortality Among Japanese Men and Women
Source: J Epidemiol. 2012 Sep 5;22(5):402–10. doi: 10.2188/jea.JE20120002 (PMC3798634; doi:10.2188/jea.JE20120002)
Supplement: Abstract in Japanese. [file je-22-402-s001.pdf]

血清α-及びγ-トコフェロール濃度と循環器疾患死亡率との関連

長尾匡則[1]、森山ゆり[2]、山岸良匡[3]、磯博康[4]、玉腰暁子[5]、the JACC Study Group

[1]獨協医科大学公衆衛生学講座、[2]前高知県衛生研究所、[3]筑波大学大学院人間総合科学研究科社会健康医学、[4]大阪大学大学院医学系研究科公衆衛生学、[5]愛知医科大学医学部公衆衛生学教室

【背景】血清トコフェロール濃度と循環器疾患死亡リスクとの関連についての研究は非常に少ない。本研究では血清α-及びγ-トコフェロール濃度と脳卒中・虚血性心疾患死亡との関連を検証することを目的とした。
【方法】文部科学省助成大規模コホート（JACC Study）の1988－1990年のベースライン調査で血清提供に同意し、脳卒中、心疾患、がんの既往がない40－79歳の男女38,158人（男性13,382人、女性24,776人）を対象として、脳卒中死亡及び虚血性心疾患死亡をアウトカムとするコホート内症例対照研究を行った。対照は、ケースと性・年齢（±5歳）・居住地域・血清保管年数がマッチした者から無作為に抽出した。
【結果】13年間の追跡期間中、脳梗塞死亡302人（男性165人、女性137人）、脳出血死亡210人（男性85人、女性125人）、虚血性心疾患死亡211人（男性114人、女性97人）が認められた。血清α-トコフェロール濃度は男性においていずれの死亡とも関連がみられなかったが、女性では全脳卒中死亡と脳出血死亡との間に負の関連がみられた。女性の血清α-トコフェロール濃度最低値群に対する最高値群の多変量調整死亡オッズ比は、全脳卒中死亡が0.35（0.16-0.77; *P* for trend = 0.009)、脳出血死亡が0.26（0.07-0.97; *P* for trend = 0.048）であった。血清γ-トコフェロール濃度は男性の脳梗塞死亡と負の関連が見られたが、女性の脳出血死亡とは正の関連がみられた。最低値群に対する最高値群、及び1標準偏差増加分における多変量調整オッズ比は、男性脳梗塞死亡が0.48（0.22–1.06; p for trend = 0.07）及び0.77（0.58–1.02)、女性脳梗塞死亡が3.10（0.95-10.12; *P* for trend = 0.052）及び1.49（1.04-2.13）であった。
【考察】女性の脳出血死亡との関連において、血清α-トコフェロール濃度は負の関連を示したが、γ-トコフェロール濃度は正の関連を示した。これらの結果にはトコフェロールの抗酸化作用と抗血栓作用が関与していることが考えられる。

キーワード：α-トコフェロール、γ-トコフェロール、ビタミンE、前向き研究、脳卒中、循環器疾患、コホート内症例対照研究
